# Characterization of an epimastigote-stage-specific hemoglobin receptor of *Trypanosoma congolense*

**DOI:** 10.1186/s13071-016-1563-9

**Published:** 2016-05-23

**Authors:** Shino Yamasaki, Keisuke Suganuma, Junya Yamagishi, Masahito Asada, Naoaki Yokoyama, Shin-ichiro Kawazu, Noboru Inoue

**Affiliations:** National Research Center for Protozoan Diseases, Obihiro University of Agriculture and Veterinary Medicine, Inada, Obihiro, Hokkaido 080-8555 Japan; Research Center for Zoonosis Control, Hokkaido University, Sapporo, Hokkaido 001-0020 Japan; Department of Protozoology, Institute of Tropical Medicine (NEKKEN), Nagasaki University, Sakamoto, Nagasaki, 852-8523 Japan

**Keywords:** *Trypanosoma congolense*, Epimastigote, Hemoglobin receptor

## Abstract

**Background:**

Since *Trypanosoma* spp*.* lack a complete heme synthesis pathway, the parasites are totally dependent on their host for heme throughout all of the stages of their life-cycle. We herein report the identification and characterization of a *T. congolense* epimastigote form (EMF)-specific hemoglobin (Hb) receptor. The gene was initially reported to encode a *T. congolense* haptoglobin (Hp)-Hb complex receptor (TcHpHbR) based on its similarity to a gene encoding a *T. brucei* Hp-Hb complex receptor (TbHpHbR).

**Methods:**

*Trypanosoma congolense* IL3000 was used in this study. A TcHpHbR gene was PCR amplified from the parasite genome. The recombinant protein was used as an immunogen to raise antibodies for immunofluorescence assay and immunoblotting. Hemoglobin uptake by the parasite was examined by using Alexa 488 labelled Hb and visualized by confocal laser scanning microscopy. The qualitative and quantitative interaction between TcHpHbR and its ligand were measured using a surface plasmon resonance assay.

**Results:**

We found that, unlike TbHpHbR, TcHpHbR was exclusively expressed in the EMF stage at RNA and protein levels. The recombinant TcHpHbR (rTcHpHbR) was co-precipitated with free-Hb in a GST-pull down assay. Surface plasmon resonance revealed that rTcHpHbR binds free-Hb with high affinity (dissociation constant (*K*_d_) = 2.1×10^-8^ M) but free-Hp with low affinity (*K*_d_ = 2.2×10^-7^ M). Furthermore, Alexa 488-labelled-Hb was only taken up by the EMF and co-localized with tomato lectin, which is a marker of endocytic compartments (flagellar pocket and lysosome).

**Conclusion:**

We conclude that the *T. congolense* EMF takes up free-Hb via TcHpHbR, a receptor which is specific to this developmental stage. We therefore propose renaming TcHpHbR as *T. congolense* EMF-specific Hb receptor (TcEpHbR).

**Electronic supplementary material:**

The online version of this article (doi:10.1186/s13071-016-1563-9) contains supplementary material, which is available to authorized users.

## Background

Many living organisms consume oxygen for energy production. Heme proteins are greatly involved in the metabolism of oxygen. Since heme proteins have essential roles in biological activities such as respiration, antioxidation and drug metabolism [1], eukaryotes are generally capable of *de novo* heme synthesis. Heme is synthesized from succinyl Co-A and glycine through eight catalytic steps and incorporated into heme proteins such as cytochrome *c* and peroxidase [2]. However, previous studies and a whole genome analysis revealed that trypanosomatids, such as *Trypanosoma* spp. and *Leishmania* spp. lack key enzymes for heme biosynthesis. The parasites therefore depend on their host as a source of heme. Hemoglobin and heme uptake have been studied in *T. cruzi*, *T. brucei* and *Leishmania* spp. *Trypanosoma cruzi* possesses an ATP binding cassette (ABC) transporter for hemoglobin uptake, whereas *Leishmania* spp. have one hemoglobin receptor and an ABC transporter [3–5]. *Trypanosoma brucei*, on the other hand, possesses a haptoglobin (Hp)-hemoglobin (Hb) complex receptor (TbHpHbR, Gene ID: Tb927.6.440), which is exclusively expressed in the blood stream form (BSF) of the parasite [6]. In mammalian blood, hemoglobin, which is released through hemolysis, binds to haptoglobin to form a complex which is immediately detoxified and taken up by macrophages for hemoglobin metabolism [7]. Thus, *T. brucei* BSF takes up the Hp-Hb complex via TbHpHbR-mediated endocytosis [6, 8, 9]. It was reported that *T. congolense*, a causative agent of nagana, which is a devastating disease of domesticated and wild animals in Africa, possessed an orthologue of the TbHpHbR gene (Gene ID: TcIL3000.10.2930, TcHpHbR), and the crystal structure of TcHpHbR protein was revealed [9]. Interestingly, an exhaustive proteome analysis suggested that, unlike TbHpHbR, TcHpHbR appeared to be exclusively expressed in the epimastigote form (EMF) of *T. congolense* [10]. The vector stages of trypanosomes, particularly the procyclic form (PCF) and EMF, appear to require a greater amount of heme than the BSF due to their fully activated cytochrome-mediated mitochondrial respiration [11, 12]. However, the mechanisms underlying heme or hemoglobin uptake in the vector stages of African trypanosomes remain to be elucidated. The tsetse fly (*Glossina* spp.), which is the sole vector of African trypanosomes, periodically ingests blood meals from mammalian hosts. Thus, it is expected that the vector stages of the parasite will be exposed to a high concentration of free-Hb in each blood meal of the tsetse fly. Based on the proteomic data showing that this molecule was expressed only in EMF of *T. congolense* [10], we therefore hypothesized that the TcHpHbR would be the EMF-specific hemoglobin receptor of the parasite. As *T. congolense* IL3000 can be grown in all of the four main life-cycle stages in vitro, we utilized this cell line to elucidate the developmental expression of TcHpHbR. Recombinant TcHpHbR (rTcHpHbR) was used to determine the ligand specificity of the receptor [13–15].

## Methods

### Trypanosomes and culture conditions

*Trypanosoma congolense* IL 3000 (TcIL3000) strain, which was isolated on the border of Kenya and Tanzania and *T. brucei brucei* GUTat 3.1 strain, which was isolated in Uganda, were used in the present study and were cultured as previously described [13]. Briefly, *T. congolense* and *T. brucei* BSFs were cultured in HMI-9 medium supplemented with 20 % fetal bovine serum (FBS) at 33 °C or 37 °C, respectively [13]. *T. congolense* PCFs and EMFs were cultured in TVM-1 medium containing 20 % FBS at 27 °C. *T. congolense* metacyclic form (MCF) were prepared from the supernatant of the medium, in which the *T. congolense* EMFs were grown, and purified using DE 52 anion-exchange column chromatography [16].

### Production of TcHpHbR and TbHpHbR proteins

Fragments of the TcHpHbR and TbHpHbR genes without the signal sequences were amplified from genomic DNAs of *T. congolense* IL3000 and *T. b. brucei* GUTat 3.1, respectively. The primers that were used in the present study are shown in Table 1. The truncated TcHpHbR and TbHpHbR genes were cloned into pET28a (Novagen Merck Millipore, Darmstadt, Germany) or pGEX6p-1 (GE Healthcare Bio-Sciences Corp., Little Chalfont, UK) plasmids to induce the expression of His- or GST-tagged proteins. The His- or GST-tagged proteins were then purified using a Ni-beads column (Cat. No. 30210, QIAGEN, Venlo, Holland) or a glutathione sepharose beads column (Cat. No. 17075601, GE Healthcare Bio-Sciences Corp.), respectively. The recombinant proteins were dialyzed against phosphate buffered saline (PBS) and concentrated to a final concentration of 1 mg/mL prior to use. The recombinant proteins were kept at -30 °C until use.

**Table 1:** The primers used in the present study

Primer name	Sequence (5′-3′)
TcHpHbR-SG F	GGATCC ^*^GCTGAAGGAGAGATCAAGGT
TcHpHbR-SG R	GCGGCCGC ^**^TGAGGATTCTGTCTCAACCT
TbHpHbR-SG F	GGATCC ^*^GCTGAGGGTTTAAAAACCAA
TbHpHbR-SG R	GCGGCCGC ^**^ACTAACCACGTCAACGGGCCT
18s rRNA F	GATCTGGTTGATTCTGCCAG
18s rRNA R	AAATGAGCCAGCTGCAGGTTC

The underlined sections indicate the sites of enzyme restriction: *Bam HI, **Not I

18s rRNA primers [17]

### Immunization

Five female 7-week-old ICR mice (CLEA Japan, Inc., Tokyo, Japan) were immunized with 50 μg (50 μl in volume) of His-tagged rTcHpHbR, which was emulsified in an equal volume of adjuvant TITERMAX® GOLD (TiterMax USA Inc., Norcross, USA). The immunizations were performed by subcutaneous injection (one primary and four booster injections) at two week intervals. Two weeks after the last booster injection, blood was collected by cardiac puncture at terminal anesthesia. Serum was prepared by centrifugation of coagulated blood at 15,000 × *g* for 1 min at room temperature. The animal experiments were performed in accordance with the standards of animal experimentations in Obihiro University of Agriculture and Veterinary Medicine (No. 27–92).

### Southern blot analysis

Genomic DNA extracted from TcIL3000 was digested with restriction enzymes *Nsi* I, *Sac* II and *Pst* I (Roche Diagnostics K. K., Tokyo, Japan). After restriction enzyme digestion, DNA (10 μg/well) was separated in a 1 % agarose gel. The electrophoretically separated DNA was transferred onto a nylon membrane (Cat. No. RPN303B, GE Healthcare Bio-Sciences, UK) and the membrane was hybridized with alkaline phosphatase-labelled DNA probes (Cat. No. RPN3680 Alkphos Direct Labeling Reagent, GE Healthcare Bio-Sciences). The DNA probes for the detection of the TcHpHbR gene were prepared by a PCR using primers shown in Table 1. For visualization, the membrane was incubated in CDP-STAR detection reagent (Cat. No. RPN3682, GE Healthcare Bio-Sciences).

### Northern blot analysis

Total RNA from TcIL3000 BSF, PCF, EMF and MCF was extracted using RNA extraction reagent (Cat. No. 15596–018, Thermo Fisher Scientific, Hudson, USA) according to the manufacturer’s instructions. Ten micrograms of total parasite RNA was separated on a 0.8 % agarose gel containing 2.2 M formaldehyde in 3-[N-morpholino] propanesulfonic acid (MOPS) buffer. The RNA was transferred onto a nylon membrane (GE Healthcare Bio-Sciences Corp.) and then fixed to the membrane by UV-induced crosslinking. The transferred RNA was probed with alkaline phosphatase-labelled DNA probes (GE Healthcare Bio-Sciences Corp.) under high-stringency conditions. The DNA probes to detect TcHpHbR mRNA and the reference transcript (18S ribosomal RNA) were prepared by a PCR using the primers shown in Table 1 [17]. Probe binding was visualized with CDP-STAR detection reagent (GE Healthcare Bio-Sciences Corp.) according to the manufacturer’s instructions.

### Western blotting

Total proteins were extracted from parasites by incubating them in cell lysis buffer (20 mM Tris–HCl pH 8.0, 150 mM NaCl, 1 mM MgCl_2_, 1 mM CaCl_2_, 10 % glycerol, 1 % Triton-X 100, protease inhibitor cocktail (Cat. No. 1836153, Roche Diagnostics K. K.)) for 4 h at 4 °C. The protein extracts (2 μg) were separated by 10 % sodium dodecyl sulfate-polyacrylamide gel electrophoresis (SDS-PAGE), and electrophoretically transferred onto a polyvinylidene difluoride membrane (Cat. No. RPN303F, GE Healthcare Bio-Sciences Corp.). Western blotting was performed as previously described [18].

### Confocal laser scanning microscopy

BSF, PCF, EMF and MCF cells were collected from culture supernatants, and washed 3 times with PBS. The cell suspensions were spread over glass slides, air-dried and fixed with methanol. The specimens were incubated with 1:100-diluted anti-rTcHpHbR mouse sera and 20 μg/ml biotinylated tomato lectin (Cat. No. B1175-1, Vector Laboratories, Burlingame, USA). After washing with PBS containing 0.05 % Tween 20 (PBS-T), the slides were incubated with 1:200-diluted anti-mouse IgG conjugated with FITC (Wako Pure Chem., Osaka, Japan) and 30 μg/ml streptavidin conjugated with fluorochrome (Cat. No. SA-5594, Vector laboratories). Nucleus and kinetoplast DNA was stained with Hoechst 33342 (Cat. No. 346–07591, Dojindo, Kumamoto, Japan). A confocal laser scanning microscope (Leica Microsystems GmbH, Wetzlar, Germany) was used to observe the prepared specimens.

### Hemoglobin and haptoglobin uptake

Red blood cells from defibrinated bovine blood were washed 3 times in PBS by centrifugation at 910 × *g* for 7 min. The red blood cells were then resuspended in PBS, diluted 10 times with sterilized distilled water to induce hemolysis and centrifuged at 15,000 × *g* for 10 min at 4 °C. In order to obtain bovine Hb powder, the supernatant was lyophilized using a freeze-drier (VD-500R, TAITEC, Saitama, Japan), and kept at -30 °C until use. Bovine Hb and commercially purchased Hp (Cat. No. 8010, Life Diagnostics Inc., West Chester, USA) were labelled with Alexa 488 (Hb ^A488^ and Hp^A488^) (Cat. No. A-10235, Thermo Fisher Scientific). Hp^A488^ Hb complex was prepared by mixing an equal volume of 2 mg/ml Hp^A488^ and 2 mg/ml Hb solution for 30 min at 37 °C. TcIL3000 PCF, EMF and MCF were incubated in TVM-1 medium with 20 μg/mL Hb^A488^, Hp^A488^ or Hp^A488^ Hb complex for 2.5 h at 27 °C, while TcIL3000 BSF parasites were incubated in HMI-9 medium with 20 μg/ml Hb^A488^, Hp^A488^ or Hp^A488^ Hb complex for 2.5 h at 33 °C. Thereafter, the parasites were placed on glass slides, air-dried and fixed with 100 % methanol for 10 min at room temperature. Nuclei and kinetoplasts were stained with Hoechst 33342 for 30 min at 37 °C. The fixed parasites were incubated with PBS containing 20 μg/ml biotinylated tomato lectin for 1 h at 37 °C. The parasites were then washed with PBS-T 3 times and incubated with PBS containing 30 μg/ml fluorochrome-labelled streptavidin for 1 h at 37 °C. A confocal laser scanning microscope (TCS-NT, Leica Microsystems GmbH) was used to observe the prepared specimens.

### GST pull-down assay

To analyze the interaction between TcHpHbR and its ligand, a GST pull-down assay was performed using GST fusion rTcHpHbR (GST-rTcHpHbR) and glutathione Sepharose beads 4B [19]. The beads and GST-rTcHpHbR were incubated with FBS diluted twice with PBS, or 2 mg/mL Hb in PBS overnight at 4 °C. After washing, the bound proteins were eluted with SDS sample buffer (125 mM Tris pH 6.8, 10 % 2-mercaptoethanol, 4 % sodium dodecyl sulfate, 10 % sucrose, 0.01 % bromophenol blue). Finally, the eluted samples were separated by 15 % SDS-PAGE and stained with Coomassie Brilliant Blue.

### Surface plasmon resonance assay

Qualitative and quantitative interactions between TcHpHbR and its ligand were measured using a surface plasmon resonance assay (SPR). SPR was performed using a Biacore X analytical system (GE healthcare Bio-Sciences Corp.). The qualitative interaction between the His-tagged recombinant proteins (rTcHpHbR, rTbHpHbR) and the analytes (free-Hb, free-Hp or HpHb complex) was measured at a flow rate of 20 μl/min. Free-Hb and free-Hp were diluted to 1 μg/ml, 10 μg/ml and 100 μg/ml with HBS-EP running buffer (Cat. No. BR100188, GE Healthcare Bio-Sciences Corp.). HpHb complexes were prepared by incubating free-Hp and free-Hb at various concentrations, namely HpHb 10–50 (Hp 10 μg/ml and Hb 50 μg/ml), HpHb 50–50 (Hp 50 μg/ml and Hb 50 μg/ml), HpHb 100–50 (Hp 100 μg/ml and Hb 50 μg/ml), HpHb 50–10 (Hp 50 μg/ml and Hb 10 μg/ml), and HpHb 50–100 (Hp 50 μg/ml and Hb 100 μg/ml). rTcHpHbR and rTbHpHbR were diluted to 100 μg/ml with 10 mM sodium acetate, pH 4.5 (Cat. No. BR100350, GE Healthcare Bio-Sciences Corp.) and coupled to the surface of CM5 sensor chips (Cat. No. BR100530, GE healthcare Bio-Sciences Corp.). The final amounts of immobilized rTcHpHbR and rTbHpHbR were 5450 resonance units (RU) and 8000 RU, respectively.

The quantitative interaction between recombinant proteins and analytes was measured at a flow rate of 30 μl/min. Free-Hb and free-Hp were serially diluted to 10, 1, 0.1, 0.01, 0.001, 0.0001 μM as analyte solutions. rTcHpHbR and rTbHpHbR were diluted to 10 μg/ml with 10 mM sodium acetate (pH 4.5) and immobilized on the CM5 sensor chips. The final amounts of immobilized rTcHpHbR and rTbHpHbR were 800 RU and 480 RU respectively. All sensorgrams were approximated to the ideal curves by non-linear curve fitting, and the association rate constant and dissociation rate constant were calculated from the approximate curves using BIAevaluation software program (BIACORE Co., Ltd. Tokyo, Japan). The dissociation constant (*K*_d_) was calculated by dividing the dissociation rate constant by the association rate constant [20].

## Results

### Cloning and the expression profile of TcHpHbR

**Fig. 1:**
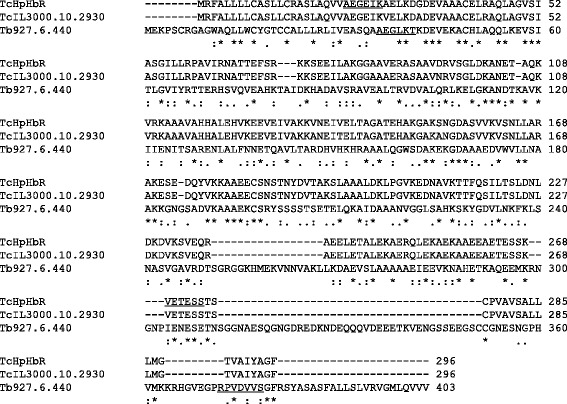
The amino acid alignment of TcHpHbR, TcIL3000.10.2930 and Tb927.6.440. The alignment of the amino acid sequences of TcHpHbR (deduced from the corresponding gene of TcIL3000), with the alignments of TcHpHbR and TbHpHbR (obtained from the TritrypDB database) as a reference. The conserved residues are shown as: fully conserved (*), strongly conserved (:) and weakly conserved (.). The underlined sections (AEGEIK and VETESS in TcHpHbR, AEGLKT and RPVDVVS in TbHpHbR) indicate the regions of the PCR primer sets that were used for recombinant protein expression

**Fig. 2:**
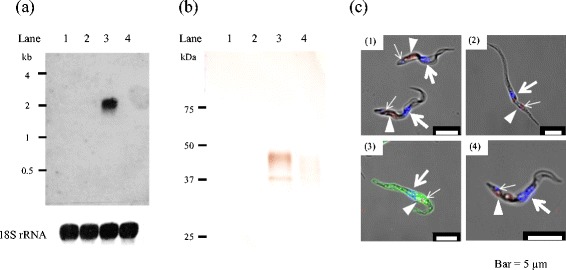
Expression profile of TcHpHbR. **a** The TcHpHbR mRNA expression profile was examined by Northern blotting. Total RNA extracted from TcIL3000 BSF (Lane 1), PCF (Lane 2), EMF (Lane 3) and MCF (Lane 4) were used for the analysis. The DNA probe to detect TcHpHbR mRNA was prepared by a PCR using the primers shown in Table 1. 18S rRNA was used as an internal reference. **b** The TcHpHbR protein expression profile was examined by Western blotting. Total proteins (400 μg/ml) extracted from TcIL3000 BSF (Lane 1), PCF (Lane 2), EMF (Lane 3) and MCF (Lane 4) were analyzed by immunoblotting using anti-rTcHpHbR mouse sera. **c** The cellular localization of TcHpHbR in TcIL3000 BSF (1), PCF (2), EMF (3) and MCF (4) was examined in an indirect immunofluorescence assay (IFA) using anti-rTcHpHbR mouse sera. The *green*, *blue* and *red* signals indicate TcHpHbR, DNA (*bold arrow*: nucleus, *fine arrow*: kinetoplast) and the endocytic compartment (*arrowhead*), respectively

The truncated TcHpHbR gene was PCR-amplified from TcIL3000 genomic DNA based on the reported sequence (Gene ID: TcIL3000.10.2930). In order to determine the copy number of the TcHpHbR gene in the TcIL3000 genome, a Southern blot analysis was performed. In single-digestions of the TcHpHbR gene with *Nsi* I and *Sac* II*,* in each case three signals (*Nsi* I : 2,700 bp, 1,800 bp, 1,300 bp, *Sac* II : 14,000 bp, 8,800 bp, 1,800 bp) were observed (Additional file 1: Figure S1, lanes 1 and 2). Since the common signal at 1,800 bp was observed by the single-digestions, the TcHpHbR gene was found to be tandemly arranged. Upon *Pst* I treatment, which cut the flanking region of the TcHpHbR gene, only two signals (3,400 bp and 15,900 bp) were observed (Additional file 1: Figure S1, lane 3). Taken together, these results indicated that the TcHpHbR gene occurs in two copies tandemly arranged in the parasite genome (Additional file 2: Figure S2). The deduced amino acid sequence of TcHpHbR displayed 30 % identity with TbHpHbR (Fig. 1). In order to examine the expression profile of TcHpHbR during the life-cycle of the parasite, Northern blotting, Western blotting and confocal laser scanning microscopy were performed. These analyses revealed that the transcription of TcHpHbR mRNA (2 kb) exclusively occurred in the EMF stage of the parasite (Fig. 2a, Lane 3). Consistent with the finding of EMF-specific mRNA transcription, TcHpHbR protein was specifically expressed as 42 kDa and 37 kDa proteins in EMF (Fig. 2b, Lane 3). Each of the parasite life-cycle stages (from in vitro cultures) were incubated with anti-TcHpHbR polyclonal antibody, and examined by confocal laser scanning microscopy. Consistent with the results of the Northern and Western blot analyses, the cell surface of the EMF stage parasite was specifically stained by the polyclonal antibody (Fig. 2 C3).

### Hemoglobin uptake in *T. congolense*

**Fig. 3:**
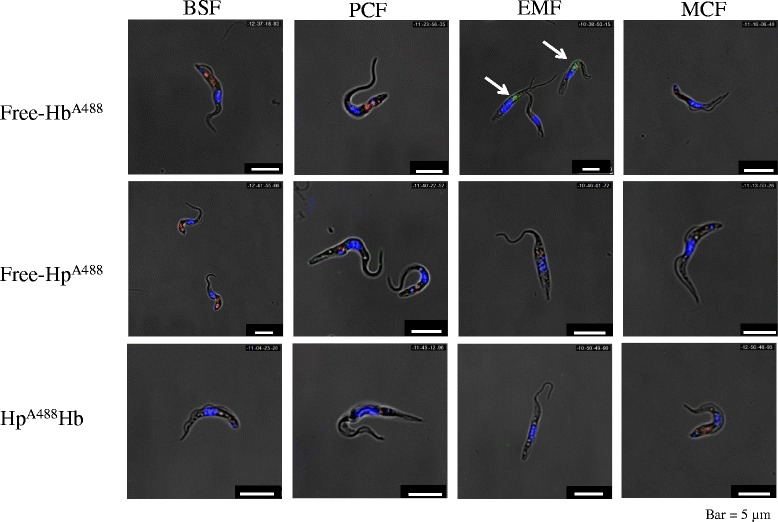
Comparison of hemoglobin uptake throughout the life-cycle stages of the parasite. The lysosomal accumulation of free-Hb^A488^, free-Hp^A488^ or Hp^A488^ Hb complex was examined throughout the stages of the TcIL3000 life-cycle*.* The green, blue and red signals indicate Alexa 488-labelled proteins, DNA (nucleus and kinetoplast) and the endocytic compartment (flagellar pocket and lysosome), respectively. The arrows indicate the accumulation of free-Hb^A488^ in the endocytic compartment in EMFs

The uptake of free-Hb^A488^, free-Hp^A488^ or Hp^A488^ Hb complex in each of the parasite life-cycle stages was examined in vitro (Fig. 3). Endocytic compartments (flagellar pocket and lysosomes) were also visualized as red spots using biotinylated tomato lectin and fluorochrome-labelled streptavidin. The EMF-specific uptake of free-Hb^A488^ was visualized as green spots indicated by arrows closed to the nucleus and kinetoplast. In addition, free-Hb^A488^ (green spot) was found to co-localize with biotinylated tomato lectin (red spot), an endocytic compartment marker, in EMF parasites (Fig. 3, EMF panel of the first row). On the other hand, no detectable free-Hp^A488^ and Hp^A488^ Hb complex uptake was observed in any of the developmental stages of the parasite (Fig. 3, panels of the second and third rows).

### Direct interaction of TcHpHbR and free-hemoglobin

**Fig. 4:**
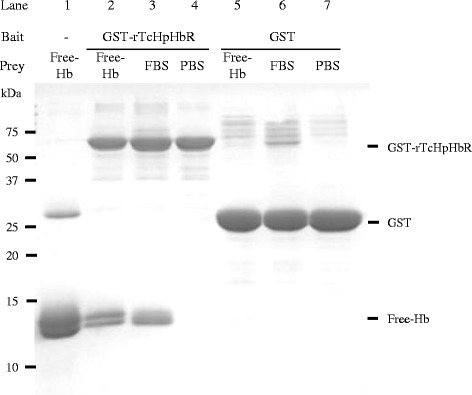
Assessment of qualitative binding between rTcHpHbR and hemoglobin by a pull-down assay. The qualitative interaction between rTcHpHbR and free-Hb was analyzed by a pull-down assay using glutathione sepharose beads. Free-Hb (Lane 1), GST-rTcHpHbR (Lane 4) and GST (Lane 7) were used as the size standards of each protein. GST-rTcHpHbR (Lanes 2–4) and GST (Lanes 5–7) were used as bait proteins. Free-Hb (Lanes 2, 5) and diluted FBS (Lanes 3, 6) were used as prey proteins

The direct interaction of TcHpHbR and free-Hb was examined by a GST pull-down assay. Free-Hb purified from bovine erythrocytes occurred as 25.8 kDa Hb dimer and as 12.8 kDa α-subunit and 13.2 kDa β-subunit (Fig. 4, Lane 1). GST-rTcHpHbR was observed to have an expected molecular mass of 64 kDa (Fig. 4, Lanes 2–4), while that of GST was 25 kDa (Fig. 4, Lanes 5–7). When GST-rTcHpHbR was incubated with 2 mg/ml free-Hb, the α- and β-subunits of Hb were co-precipitated (Fig. 4, Lane 2). In addition, a 12.8 kDa α-subunit and a 13.0 kDa γ-subunit of fetal Hb were co-precipitated when GST-rTcHpHbR was incubated with diluted FBS containing 0.09 mg/ml fetal Hb (Fig. 4, Lane 3). In contrast, GST did not interact with Hb (Fig. 4, Lanes 5–6).

### Binding parameters of TcHpHbR

**Fig. 5:**
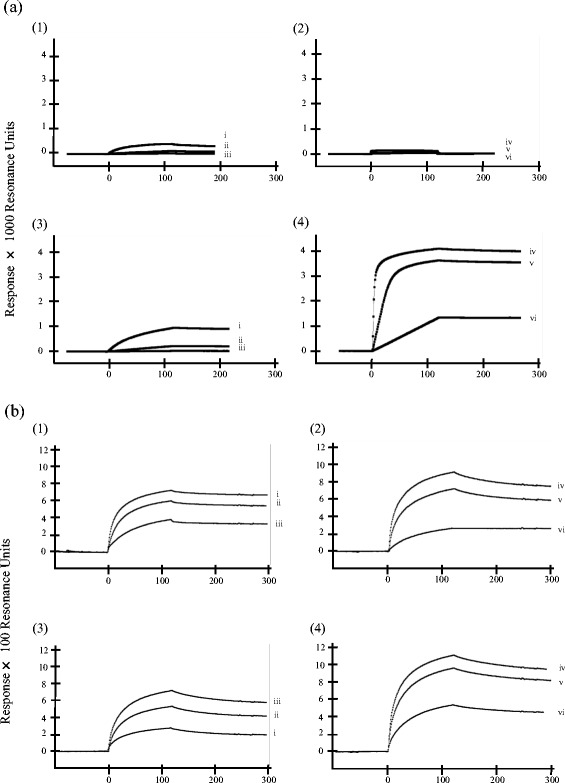
SPR assay of rTcHpHbR and rTbHpHbR. The quantitative binding assay of rTcHpHbR and rTbHpHbR was performed using an SPR assay. The vertical axis shows the binding response, while the horizontal axis shows the running time (in seconds). His-tagged rTcHpHbR or rTbHpHbR were used as immobilized receptor. **a** The interaction between rTbHpHbR and free-Hp (1), rTbHpHbR and free-Hb (2), rTcHpHbR and free-Hp (3), rTcHpHbR and free-Hb (4). The concentrations of used analytes were as follows: free-Hp 100 μg/ml (i), 10 μg/ml (ii), 1 μg/ml (iii) and free-Hb 100 μg/ml (iv), 10 μg/ml (v), 1 μg/ml (vi). **b** The interaction between the HpHb complex and rTbHpHbR (1 and 2) or rTcHpHbR (3 and 4). The concentrations of used analytes were as follows: Hp 100 μg/ml and Hb 50 μg/ml (i), Hp 50 μg/ml and Hb 50 μg/ml (ii and v), Hp 10 μg/ml and Hb 50 μg/ml (iii), Hp 50 μg/ml and Hb 100 μg/ml (iv), Hp 50 μg/ml and Hb 10 μg/ml (vi)

To compare the binding affinity of TbHpHbR with that of TcHpHbR, an SPR assay was performed. The results showed that rTbHpHbR had a low affinity for both free-Hp (*K*_d_ = 1.8 μM) and free-Hb (*K*_d_ = 5.3 μM) (Fig. 5, a1 and a2). In contrast, rTcHpHbR displayed high affinity for both free-Hp and free-Hb (Fig. 5, a3 and a4). However the affinity of rTcHpHbR for free-Hb (*K*_d_ = 20.5 nM) was ten times higher than that for free-Hp (*K*_d_ = 220 nM). These results indicated that free-Hb is possibly a specific ligand for rTcHpHbR, while neither free-Hb nor free-Hp were specific ligands for rTbHpHbR. Consistent with previous reports, results indicated that TbHpHbR displayed a high affinity for HpHb complex, because the resonance units (RUs) increased in proportion to the amount of free-Hp against free-Hb. (Fig. 5, b1). In other words, when the amount of free-Hp against free-Hb was increased, the amount of HpHb complex that was bound was also increased. In contrast, for rTcHpHbR the RUs decreased inversely proportionally to the amount of free-Hp against free-Hb (Fig. 5, b3) indicating that the amount of free-Hb bound was decreased because of the increased amount of HpHb complex. The RUs of both TbHpHbR and TcHpHbR increased proportionally to the amount of free-Hb against free-Hp (Fig. 5, b2 and b4). In this case, the amounts of both HpHb complex and free-Hb were increased and these ligands interacted with TbHpHbR and TcHpHbR, respectively.

## Discussion

Unlike other eukaryotes, trypanosomes obtain heme sources extracellularly as they lack a pathway for heme synthesis [21]. As seen in *Trypanosoma* and *Leishmania,* heme uptake is important for the growth and development of the parasite [6, 22]. For example, TbHpHbR knockout mutants caused a decrease in the growth of *T. brucei* in mice [6]. In contrast to the mammalian life-cycle stage, the vector life-cycle stages of trypanosomes require much higher amounts of heme as they need to produce heme proteins for an active electron transport chain [23]. Thus hemoglobin uptake via hemoglobin receptors is essential for the survival of the vector life-cycle stages [22]. Nevertheless, the mechanisms by which the vector life-cycle stages of African trypanosomes, such as the PCF and EMF, take up heme has not been well examined.

In the present study, we examined the expression profile, binding specificity and binding parameters of TcHpHbR, which was previously reported as a *T. congolense* orthologue of TbHpHbR [6, 8, 9]. Although TcHpHbR was described as a single copy gene, we showed that TcHpHbR was a 2 copy-gene with a tandem arrangement (Additional file 1: Figure S1 and Additional file 2: Figure S2). The amino acid sequences of TcHpHbR and TbHpHbR indicated that they shared 30 % identity (Fig. 1) [6]. Higgins et al. reported that TcHpHbR was structurally similar to two well-characterized trypanosome GPI-anchored surface proteins (namely, VSG MITat 1.2 and GARP) in terms of their characteristic three-helical bundle [9]. Nevertheless, TcHpHbR was exclusively transcribed and translated as 37 kDa and 42 kDa proteins in the EMF of *T. congolense* (Fig. 2). Presumably, the 37 kDa protein was unmodified TcHpHbR without the N-terminal signal peptide (Met_1_ to Val_37_), while the 42 kDa protein was post-translationally modified TcHpHbR. The molecular mass of TcHpHbR was similar to the predicted molecular mass of TbHpHbR (43.3 kDa), the apparent mass of which was 72 kDa because of N-glycosylation [6]. Thus TcHpHbR appeared to have fewer post-translational modifications than TbHpHbR. This might be related to their different expression profiles during the parasite life-cycle. It was reported that TcHpHbR was structurally truncated in comparison to TbHpHbR [9], presumably because it does not need to protrude above a VSG layer, which is absent on the cell surface of EMF cells. Unlike TbHpHbR, the cellular localization of TcHpHbR was not limited to the flagellar pocket, rather it was found throughout the entire surface of EMF cells (Fig. 2c). However, since the uptake of Hb only occurred through the flagellar pocket (Fig. 3), cell surface TcHpHbR seemed to translocate to the flagellar pocket and to be endocytosed when it has bound Hb. This might suggest the presence of unique mechanisms that underlie the translocation of cell surface receptors to the flagella pocket in EMF. As we expected, the ligand specificity of TcHpHbR was also different from that of TbHpHbR. TbHpHbR is an HpHb complex-specific receptor [6], whereas TcHpHbR binds free-Hb with high affinity (Figs. 4 and 5a(4)). The ligand binding characteristics of TcHpHbR and TbHpHbR differed when the amount of free-Hp was exchanged for a specific amount of free-Hb (50 μg/ml) (Fig. 5b). According to this result, we speculated that the majority of free-Hb molecules could not form the HpHb complex in the tsetse midgut due to the lack of a sufficient amount of Hp molecules. Thus, the different ligand specificity of TcHpHbR and TbHpHbR may have evolved as a consequence of the adaptation of the different life-cycle stages of the two species to their different habitat within their vector. Since *T. congolense* EMFs occupy the proboscis of the tsetse fly vector, it seems that they are periodically exposed to a high level of free-Hb during blood meals. Hence, *T. congolense* EMFs may effectively take up free-Hb by the specific receptor, TcHpHbR. In contrast, *T. brucei* EMFs inhabit the salivary glands of their tsetse fly vector. Thus, the parasites do not come into contact with free-Hb.

## Conclusion

We found that TcHpHbR, a TbHpHbR orthologue in *T. congolense*, was EMF-specific free-Hb receptor. We therefore propose that TcHpHbR should be renamed as *T. congolense* epimastigote-specific free-Hb receptor (TcEpHbR).

## Additional files

Additional file 1: Figure S1. The Southern blot analysis of the TcHpHbR gene. TcIL3000 genomic DNA treated with *Nsi*I (lane 1), *Sac*II (lane 2), *Pst*I (lane 3) or neat TcIL3000 genomic DNA (lane 4) was subjected to Southern blotting. (PPTX 1.18 mb) Additional file 2: Figure S2. The genome organization and a restriction map of the TcHpHbR gene. (PPTX 40.0 kb)
