# Supplementary figures and images for: Characterization of an epimastigote-stage-specific hemoglobin receptor of Trypanosoma congolense
Source: Parasit Vectors. 2016 May 23;9:299. doi: 10.1186/s13071-016-1563-9 (PMC4877808; doi:10.1186/s13071-016-1563-9)

## Slide 1
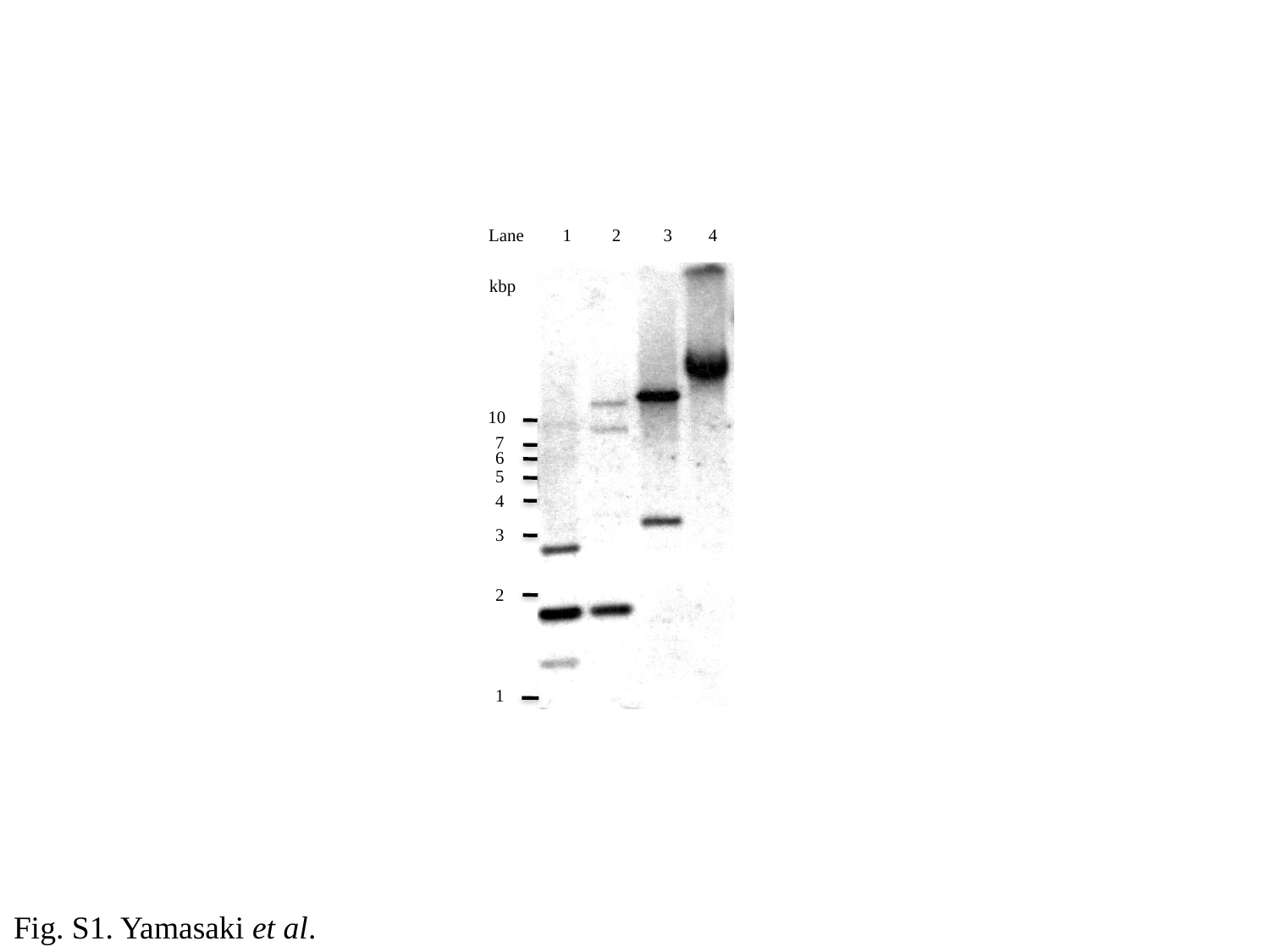

Lane
1
2
3
4
kbp
10
7
6
5
4
3
2
1
Fig. S1. Yamasaki et al.

Supplement: Additional file 1: Figure S1. — The Southern blot analysis of the TcHpHbR gene. TcIL3000 genomic DNA treated with NsiI (lane 1), SacII (lane 2), PstI (lane 3) or neat TcIL3000 genomic DNA (lane 4) was subjected to Southern blotting. (PPTX 1.18 mb) [file 13071_2016_1563_MOESM1_ESM.pptx]

## Slide 1
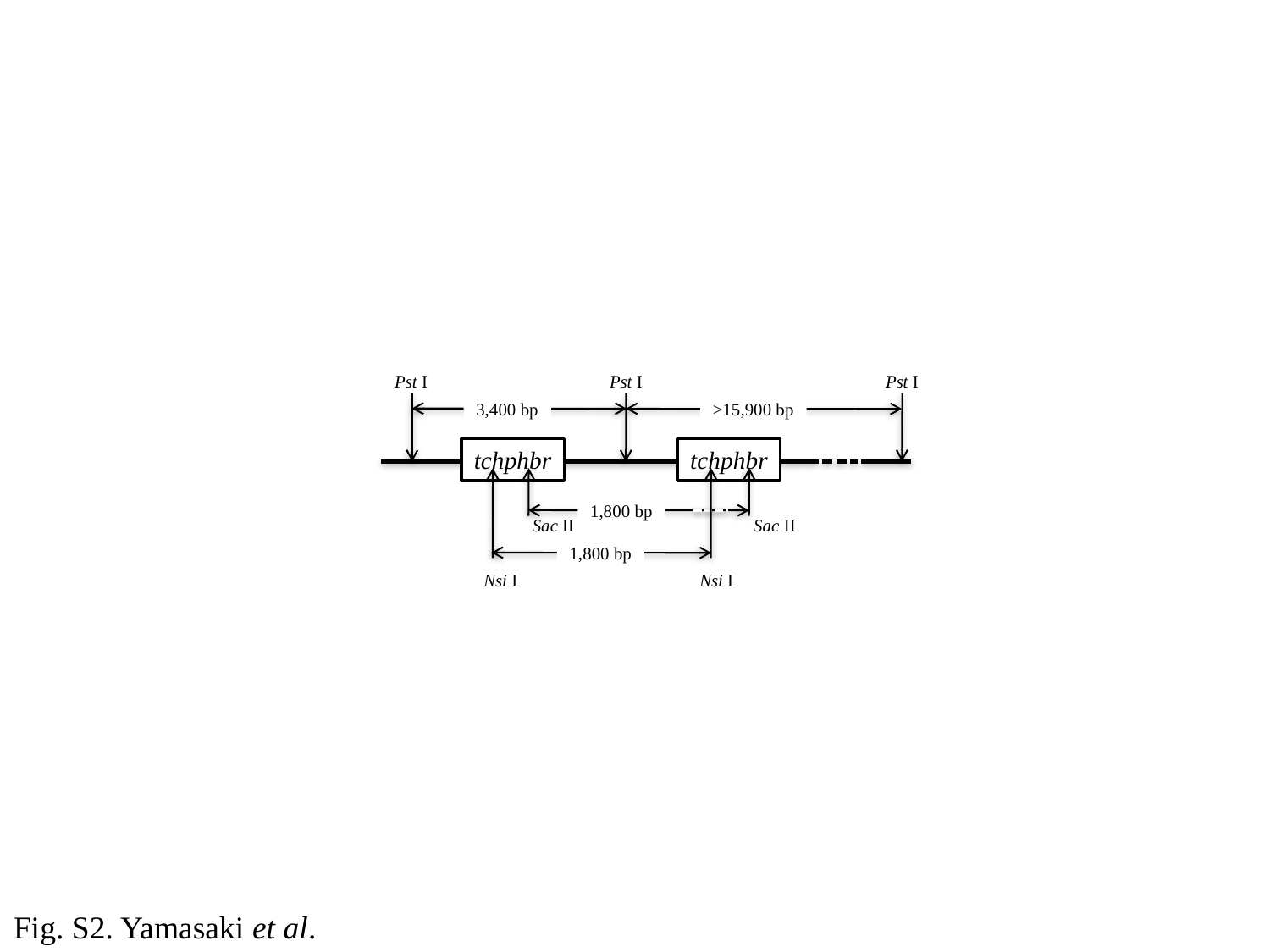

Pst I
Pst I
3,400 bp
tchphbr
Sac II
Nsi I
Pst I
>15,900 bp
tchphbr
1,800 bp
Sac II
1,800 bp
Nsi I
Fig. S2. Yamasaki et al.

Supplement: Additional file 2: Figure S2. — The genome organization and a restriction map of the TcHpHbR gene. (PPTX 40.0 kb) [file 13071_2016_1563_MOESM2_ESM.pptx]
